# Stroke unit capacity should not rely on shortening length of stay alone

**DOI:** 10.1093/esj/aakag019

**Published:** 2026-03-23

**Authors:** Giovanni Merlino, Gian Luigi Gigli, Mariarosaria Valente

**Affiliations:** SOSD Stroke Unit, Department of Head, Neck and Neurosciences, Udine University Hospital, Udine, Italy; Clinical Neurology, Department of Medicine (DMED), University of Udine, Udine, Italy; Clinical Neurology, Department of Medicine (DMED), University of Udine, Udine, Italy

**Keywords:** capacity, length of stay, stroke unit

Dear Editor,

We read with great interest the recent MAPSTROKE analysis by Nicolini et al. addressing access to reperfusion therapies and stroke unit (SU) capacity in Italy. The study provides an important national perspective and clearly identifies a key bottleneck in stroke systems of care: despite adequate geographical access to reperfusion therapies, current SU capacity appears insufficient to meet recommended targets.^1^

The authors highlight that reducing SU length of stay (LOS) could increase bed turnover and therefore improve effective SU capacity, while appropriately emphasising that such strategies must be carefully evaluated to ensure that reducing LOS does not compromise access to dedicated stroke care.^1^ We believe this crucial point deserves further consideration.

A large body of evidence consistently demonstrates that organised SU care improves survival, independence and long-term outcomes.^2^ In addition, real-world data suggest that not only admission to a SU but also the proportion of time spent within it is a key determinant of outcome. Patients who spend at least 90% of their hospital stay in a SU experience fewer severe complications and are less likely to require institutional care, even when overall hospital stay is shorter.^3^ These findings reinforce the concept that sustained exposure to organised SU care represents a major quality indicator.

At the same time, health-system pressures may directly influence access to SU care. Registry data indicate that hospital bed occupancy is associated with a reduced likelihood of SU admission, highlighting how capacity constraints can negatively affect the quality of acute stroke care.^4^ Importantly, the MAPSTROKE analysis demonstrates that current SU capacity is already insufficient to cover the national burden of stroke, indicating minimal surge capacity within the system.^1^ Together, these observations raise an important concern: shortening LOS is beneficial when it reflects clinical stability and transition to structured post-acute pathways, but reducing time in SU care because of bed pressure may lead to premature transitions of care and reduced exposure to specialised monitoring and multidisciplinary management. This is particularly relevant given the strong association between high-quality acute stroke care and improved long-term survival and quality of life.^5^

A further methodological consideration relates to the assumption of an “ideal” LOS used in modelling studies. In the MAPSTROKE analysis, the SU bed gap was recalculated assuming an ideal LOS of 3.65 days derived from the European Stroke Organisation benchmark of 100 strokes per SU bed per year, while the observed median LOS in SUs was approximately 5 days.^1^ Length of stay should not be interpreted solely as a modifiable efficiency parameter, as the difference between observed and “ideal” LOS may reflect a combination of downstream organisational factors and genuine clinical needs, which modelling studies cannot disentangle. Patients may remain longer in SUs not only because of downstream organisational bottlenecks but also because they continue to require specialised stroke care.

For these reasons, SU capacity cannot be safely expanded by shortening LOS alone. Sustainable improvements require strengthening the entire stroke care pathway, including step-down care, early rehabilitation and early supported discharge services. In parallel, sufficient SU bed availability is essential not only to ensure admission of at least 90% of patients with stroke but also to allow prolonged monitoring and specialised care for those who continue to require it. Integrating geospatial planning with pathway-level organisational strategies represents the next crucial step for stroke system optimisation.
